# Rapid degradation of zinc oxide nanoparticles by phosphate ions

**DOI:** 10.3762/bjnano.5.209

**Published:** 2014-11-05

**Authors:** Rudolf Herrmann, F Javier García-García, Armin Reller

**Affiliations:** 1 Institut für Physik, Universität Augsburg, Universitätsstr. 1, 86159 Augsburg, Germany; 2 ICTS Centro Nacional de Microscopía Electrónica, Facultad de Químicas, Av. Complutense s/n, 28040 Madrid, Spain

**Keywords:** degradation, phosphate, silica shell, zinc oxide nanoparticles, zinc phosphate

## Abstract

Zinc oxide nanoparticles are highly sensitive towards phosphate ions even at pH 7. Buffer solutions and cell culture media containing phosphate ions are able to destroy ZnO nanoparticles within a time span from less than one hour to one day. The driving force of the reaction is the formation of zinc phosphate of very low solubility. The morphology of the zinc oxide particles has only a minor influence on the kinetics of this reaction. Surface properties related to different production methods and the presence and absence of labelling with a perylene fluorescent dye are more important. Particles prepared under acidic conditions are more resistant than those obtained in basic or neutral reaction medium. Surprisingly, the presence of a SiO_2_ coating does not impede the degradation of the ZnO core. In contrast to phosphate ions, β-glycerophosphate does not damage the ZnO nanoparticles. These findings should be taken into account when assessing the biological effects or the toxicology of zinc oxide nanoparticles.

## Introduction

Crystalline nanoparticles of the semiconductor zinc oxide (ZnO-NP) show a broad fluorescence band in the visible range when excited in the UV region [1]. During first tests of ZnO-NP for their interaction with biological systems in the SPP 1313 priority programme (*BioNanoResponses*) we observed that this fluorescence is rapidly (within seconds) quenched in some cell culture media. Tracing the effect back to its roots revealed that the presence of phosphate ions in the media is responsible for the quenching. We now wish to present results which show that the interaction with phosphate ions is not limited to the surface of the nanoparticles but finally leads to their complete destruction.

It is well known that zinc oxide can react with phosphoric acid to form various zinc phosphates which have applications, e.g., as dental cement [2]. Zinc phosphate can also be used as corrosion inhibitor for metals as it forms a protecting layer on metal surfaces [3]. This usage is possible because of the low solubility of tertiary zinc phosphate (solubility product for Zn_3_(PO_4_)_2_: ca. 10^−34^ (mol/L)^5^ [4]. However, it is often necessary to ensure tight coatings by additives [5].

The reactivity of bulk zinc oxide with phosphate ions has formerly been studied mainly from a geological point of view (alkaline solution, high temperature) [6–7]. Particles between 200 and 400 nm react with orthophosphate and polyphosphate ions under neutral and alkaline conditions under partial dissolution resulting in the formation of zinc phosphates at the surface [8].

Since ZnO-NP are used technically, e.g., as UV blockers in cosmetics, studies on interactions with biological systems are necessary. Cell culture media contain varying amounts of phosphate ions, from low (less than 100 mg/L, as in human blood) to high (more than 1000 mg/L for CO_2_-independent media). One can therefore expect that the choice of the cell culture medium is of crucial importance for biological studies of ZnO-NP. In this context, the effect of water itself must be distinguished from that of the phosphate ions. Aggregation of ZnO nanoparticles in water was attributed to partial dissolution [9]. Morphological changes of ZnO nanocrystals under the influence of atmospheric water have been reported [10]. We therefore tested the influence of pure water on size and morphology the ZnO-NP always in parallel with the reactions of phosphate buffers.

The toxicity of zinc oxide was found not to be related to particle size since toxic effects in water dispersions are due to Zn^2+^ ions whose concentration is almost equal in any sample within three days at pH 7.6 [11]. However, another investigation showed a higher toxicity for smaller particles [12]. In addition, the toxicity to marine organism was found to be shape-dependent [13]. Toxicity seems to parallel Zn^2+^ concentration which in turn is determined by the thermodynamics and kinetics of the ZnO-NP dissolution [14]. Newer results have shown that ZnO-NP can survive the cell culture media used for the studies under certain conditions, and are dissolved inside the cells after uptake. Most of their toxicity is then due to the increased Zn^2+^ concentration in the cells and formation of zinc complexes by molecular ligands [15]. Concerning dissolution kinetics of ZnO in aqueous media, no clear picture can be derived from the available data. While some find a size dependence [16–17], others do not [11,18–19]. The initial concentration of the ZnO-NP is reported to be important by some [20] but to be negligible by others [13]. For ZnO there is a high probability that different phosphate contents in the media used for the investigations significantly influence the results since a lower phosphate concentration increases the lifetime of the ZnO particles but at the same time allows for higher Zn^2+^ concentrations. That the dissolution of ZnO-NP depends on the medium is generally agreed [19,21–22]. The complexity of dissolution processes on several NP types in media, buffers and water has been stressed recently [23]. Special attention to the effects of phosphate around neutral pH has been payed only very recently [24].

The photocatalytic activity of ZnO nanoparticles can be significantly reduced by coating with SiO_2_ [25]. This is important when ZnO-NP are applied as UV blockers. We therefore also included ZnO-NP coated with silica in our tests.

## Results and Discussion

**Figure 1:**
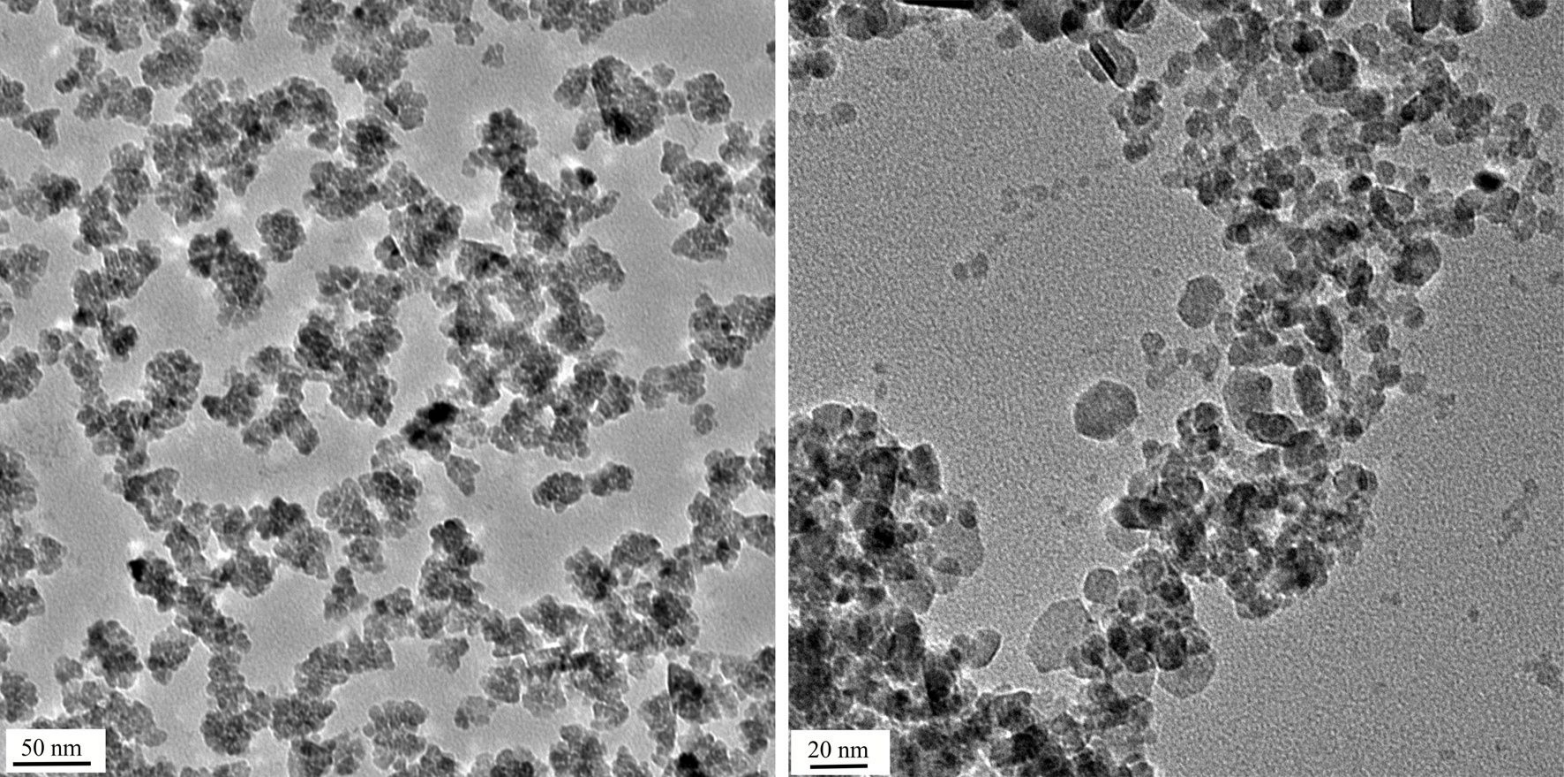
Left: ZnO-NP prepared by the acidic procedure (sample **1**, without labelling; scale bar: 50 nm). Right: ZnO NP prepared under alkaline conditions (sample **2**, without labelling; scale bar: 20 nm).

**Figure 2:**
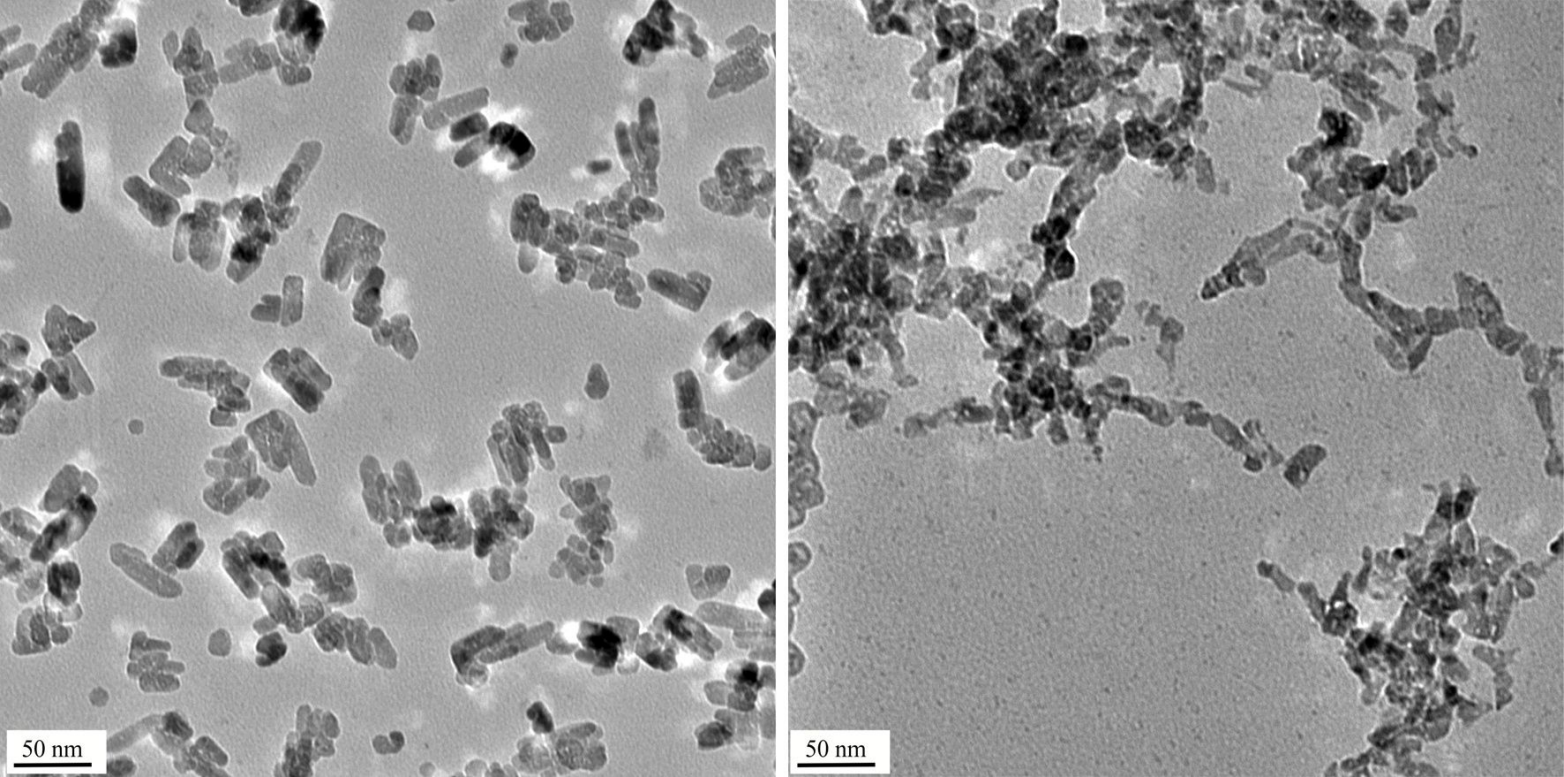
TEM pictures of ZnO-NP **1** (labeled with a perylene dye) as prepared (left) and after one hour of reaction with buffer A (right). The scale bars are 50 nm.

We tested ZnO-NP both in their native form and labelled at the surface with *N*-(2,5-bis(dimethylethyl)phenyl)-*N*'-(3-(triethoxysilyl)propyl)perylene-3,4,9,10-tetracarboxylic acid diimide (MDPI). This fluorescent dye is a convenient label for particles used in biomedical applications [26]. Similar perylene-derived dyes were applied as sensitizers for zinc oxide solar cells [27]. ZnO samples **1** were prepared by a wet method under acidic conditions [28]. In the absence of the fluorescence marker we obtained almost spherical particles of 5–10 nm diameter clustering into small heaps (Figure 1, left). The presence of the fluorescence marker changes the morphology considerably and leads to rods and (approximate) spheres (Figure 2, left). DLS measurements show that the average size of the agglomerates is the same for both samples (400 nm) in water. Samples **2** were obtained by a wet method under basic conditions [29] leading to particles of spherical and hexagonal shape with diameters in the range of 5–25 nm (Figure 1, right). The presence or absence of the fluorescent dye did not have a significant effect on the morphology. The tendency to form agglomerates in water is higher for the labelled sample (DLS size 144 and 309 nm, respectively). Sample **3** was commercial ZnO prepared by gas phase oxidation of zinc at high temperature, the so-called French process. It is a highly irregular mixture of particles with very different morphologies and sizes [30]. DLS measurements suggest similar agglomeration for labelled and unlabelled particles in water although DLS is not that reliable for such irregular particles since it was developed for spheres. All nanoparticles studied are composed of crystalline ZnO (zincite) phase, as confirmed by XRD.

Two buffers of the Sørensen type at pH 7.0 were applied. The concentration of phosphate was kept to 100 mg/L, resembling the value in human blood (buffer A), or to 2000 mg/L, similar to the value found in typical CO_2_-independent cell culture media (buffer B). For comparison, a solution of disodium β-glycerophosphate (2000 mg/L) was also used. After the reaction time (1 h or 24 h) all solid material was collected by centrifugation and subsequently washed three times with water to remove any soluble phosphate. The solid was then redispersed in ethanol and deposited on the copper grid for the subsequent TEM investigation. An aliquot was dissolved in acid and analyzed for the phophorus/zinc molar ratio by ICP-OES. The results are shown in Table 1. For pure ZnO the ratio is zero, and for pure tertiary zinc phosphate 2:3 is expected. A higher value indicates the presence of secondary zinc phosphate ZnHPO_4_ which generally precipitates faster than tertiary zinc phosphate despite its higher solubility [31]. Any intermediate value lower than 0.67 reflects a partial degradation of the ZnO-NP. The error in the determination of the ratio is in the range of 10%. For phosphorus concentrations lower than 1 mg/L (very low conversion) the ICP-OES results are considered not reliable and therefore indicated as not determinable (n.d., i.e., close to zero) in the table.

**Table 1:** Molar ratio P/Zn after one hour or one day of action by buffer solution at pH 7.0 on zinc oxide nanoparticles („+“: with fluorescence label, „−“ without). Buffer A: 100 mg/L of phosphate, buffer B: 2000 mg/L of phosphate.

sample	fluorescence label	buffer	P/Zn (1 h)	P/Zn (24 h)

1	−	A	n.d.	0.59
1	+	A	n.d.	0.72
1	−	B	0.37	0.76
1	+	B	0.23	0.66
2	−	A	0.56	0.52
2	+	A	0.61	0.60
2	−	B	0.76	0.72
2	+	B	0.62	0.72
3	−	A	0.64	0.79
3	+	A	0.63	0.66
3	−	B	n.d.	0.71
3	+	B	0.71	0.74

**Figure 3:**
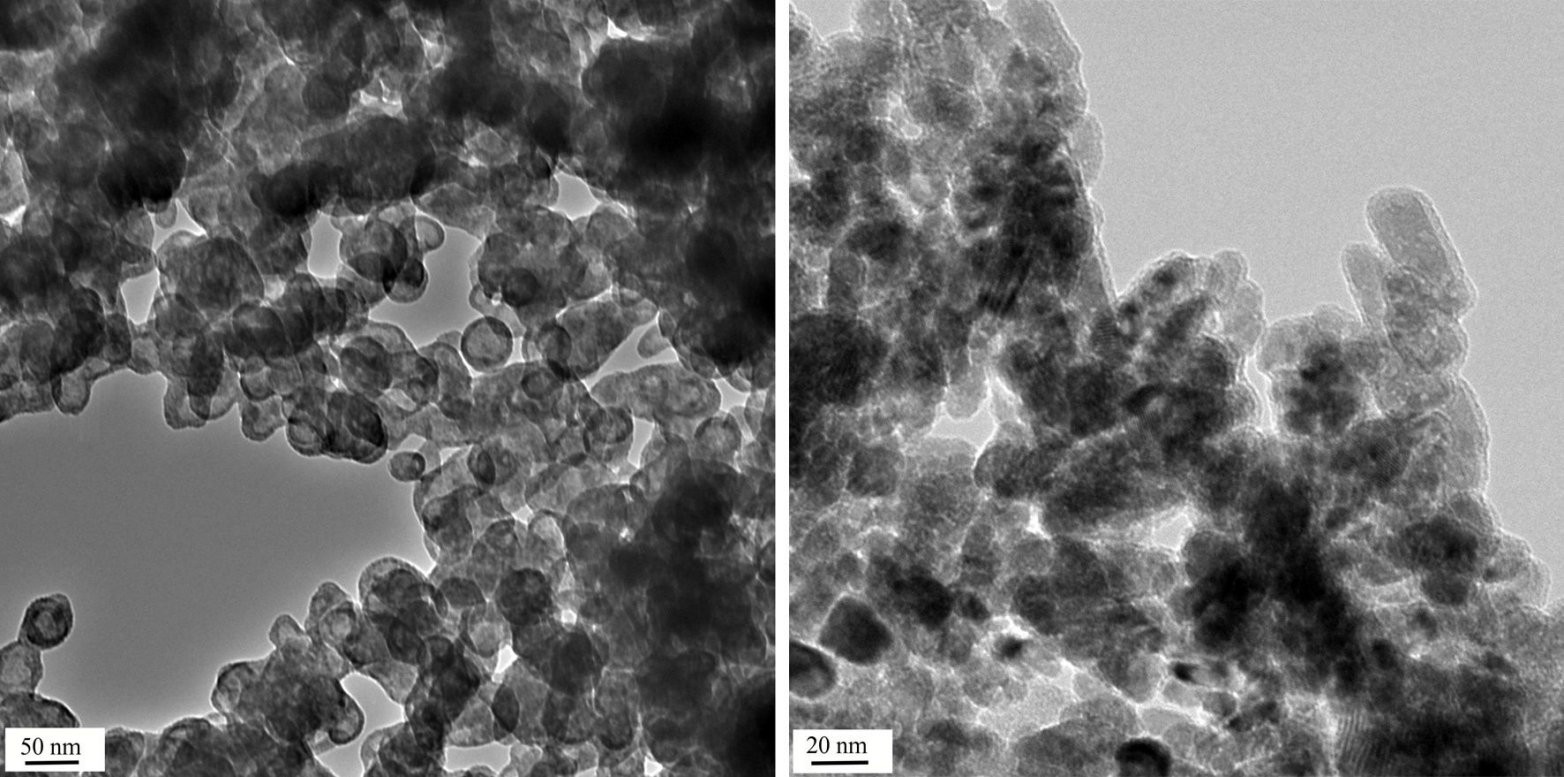
Left: TEM picture of degraded ZnO-NP **2** (labelled) after 1 hour of reaction with buffer B. The scale bar is 50nm. Right: Silica-coated ZnO-NP (sample **5**). Shell thickness: 4–5 nm. The scale bar is 20 nm.

The results clearly indicate that the degradation of the ZnO-NP is almost complete for all of the samples after 24 h. Some differences can, however, be observed after one hour of action of the buffer solutions. Thus, the particles in sample **1** (prepared under acidic conditions) are considerably more stable and only partially destroyed while there is almost no difference between one hour and one day for the particles in the other samples. As expected, buffer A (100 mg phosphate ions/L) shows a somewhat lower reactivity than buffer B (2000 mg/L). This allows one, in the case of the sample **1,** to observe the initial stage of the attack of the phosphate ions by TEM. Figure 2 shows the difference between the initial state (left) and after one hour of action of buffer A (right). The initially well-defined crystals (mainly rods and spheres) lose their shape and show a lower crystallinity at higher resolution of the TEM. They start to agglomerate strongly, a fact which we attribute mainly to the presence of amorphous zinc phosphate which acts as a glue for the remaining crystalline ZnO particles. Increasing agglomeration accompanying the partial dissolution of ZnO-NP in phosphate-free buffers has been reported [32]. After 24 h, all samples are of the same appearance. Only amorphous zinc phosphate forming approximately spherical particles with a high degree of agglomeration is observed, and the original shape of the ZnO-NP is not reflected in that of the final products. XRD measurements after a 3 h treatment of the nanoparticles with buffer B confirm that the zinc phosphate formed is indeed amorphous, the only crystalline material in the samples being residual ZnO. Note that sample **2** has already reached its final state after one hour (Figure 3, left). The precipitation of mainly amorphous zinc phosphate from solutions of zinc chloride and sodium phosphate in various cell culture media parallels this observation [33]. During the course of the reaction of buffer B with the nanoparticles for one hour, the ζ-potential of all particle dispersions has a tendency to shift to more negative values, and the hydrodynamic diameter decreases (Table 2). Due to the highly irregular shape of the zinc phosphate particles and their varying agglomeration, no further conclusions can be drawn from these measurements.

**Table 2:** Change in ζ-potential and hydrodynamic diameter after 1 h of action by buffer B (2000 mg/L of phosphate) on the nanoparticles.

sample	fluorescence label	ζ (mV) 0 h	ζ (mV) 1 h	*r* (nm) 0 h	*r* (nm) 1 h

1	−	−39.1	−44.7	344	236
1	+	−46.1	−50.2	427	408
2	−	−62.4	−67.9	262	238
2	+	−66.1	−77.0	243	161
3	+	−53.3	−74.3	455	368
4	+	−58.3	−56.7	478	382
5	+	−44.2	−49.0	990	470

**Figure 4:**
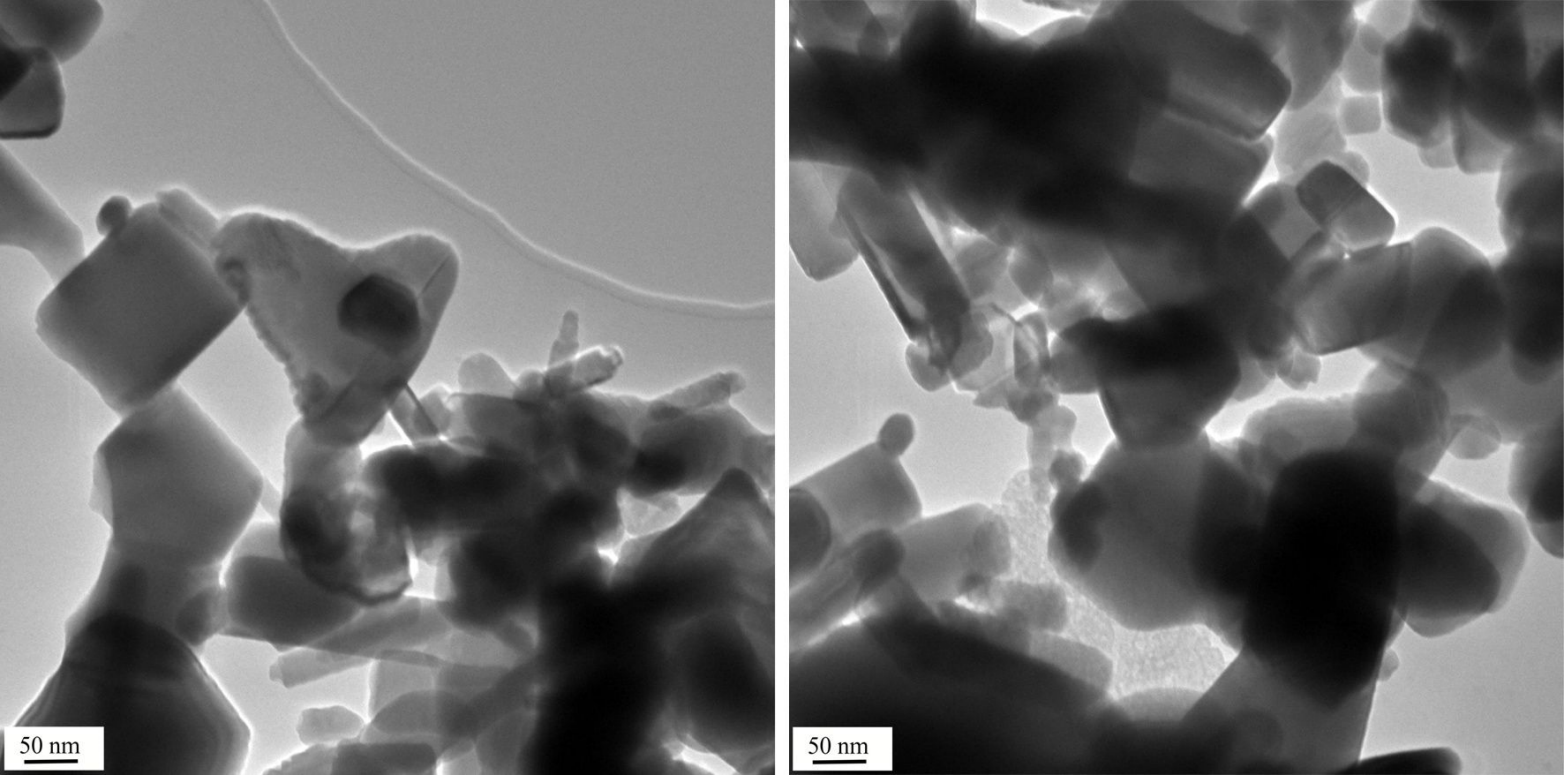
Commercial ZnO sample **3** after treatment with buffer B (2000 mg/L) for one hour. The general appearance is still that of the starting material, but signs of beginning degradation can be seen at the margin of the central triangular particle (left), and amorphous zinc phosphate starts to appear in the lower part of the right picture. The scale bars are 50 nm.

Sample **3** is an exception to the general rule that lower phosphate concentration leads to slower destruction. When used without fluorescence label there is not much reaction with buffer B (2000 mg/L phosphate) but complete degradation with buffer A (100 mg/L) after one hour. However, after one day destruction is also complete with buffer B. This behaviour can be explained in terms of the formation of a relatively compact layer of sparingly soluble zinc phosphate at the surface, as in technical corrosion protection, which is induced by the high phosphate concentration. But the protection layer is not completely compact so that after one day degradation is also complete. TEM pictures (Figure 4) show that the general appearance is still that of the starting material after one hour but signs of beginning destruction such as loss of the smooth surface and formation of agglomerates of amorphous zinc phosphate can already be seen. The zinc phosphate layer itself is not thick enough to be detected directly by TEM. Surface modification by labelling with the perylene fluorescence dye prevents the formation of a comparatively tight layer of zinc phosphate which leads to an almost complete degradation after one hour although few crystalline ZnO particles inside the amorphous agglomerate can still be observed by TEM at this point. This is no longer the case after one day.

During the etching of ZnO nanostructures with phosphate-containing solutions [34] it was found that structures obtained by thermal evaporation (such as sample **3**) generally dissolved slower than those from hydrothermal processes. This observation is only partially applicable to the nanoparticles studied. Within one day all ZnO-NP were completely destroyed by the phosphate buffers.

Photocatalytic activity of ZnO-NP, e.g., in oxidation reactions, is an unwanted feature when they are to be applied as UV blockers. Among the suggested coatings, which should block this activity, silica is mostly used in commercial preparations [25]. In principle, coatings should also prevent degradation by phosphate provided that the coating is sufficiently tight. We therefore extended our tests to two samples of coated ZnO-NP, one being a commercial sample (Maxlight ZS-64) used as obtained and labelled with the fluorescence dye (samples **4**), while the other sample was prepared by coating of labelled ZnO-NP of sample **1** (prepared under acidic conditions) with a modified Stöber procedure [35] (sample **5**).

**Figure 5:**
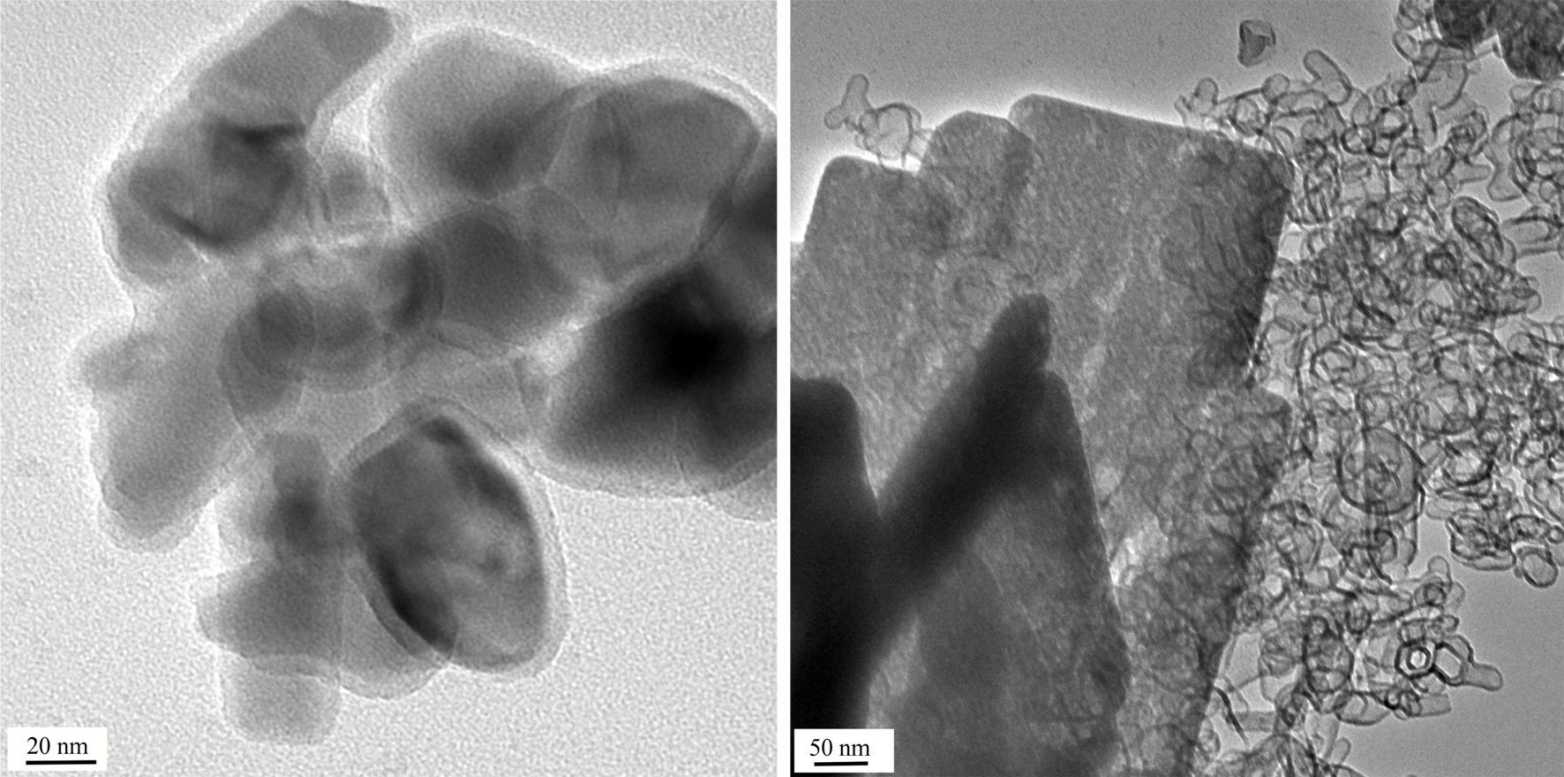
SiO_2_-coated labelled ZnO-NP (sample **4**; shell thickness 3–6 nm) before (left; scale bar 20 nm) and after (right; scale bar 50 nm) 24 h degradation reaction with buffer B.

It turned out, however, that the coating could not prevent degradation by phosphate. After 24 h the degradation was complete in all cases. The reaction rates were similar, regardless if the particles were used as received or labelled (samples **4**, Figure 5, left), or if they were produced by a different process (sample **5**). The zinc phosphate formed during the degradation process did not appear as amorphous material as in the tests with non-coated ZnO-NP but as large crystals. The silica remained as heaps of empty shells clearly separated from the crystals (Figure 5, right). The separation of the two components was confirmed by EDX analysis of the two regions: the left regions (large crystals) shows zinc and phosphorus but no silicon while the right region (empty shells) shows silicon but no zinc or phosphorus. The degradation of the zinc oxide core of the coated particles is therefore complete. The silica shell (thickness 3–6 nm) is obviously not sufficiently compact to prevent the access of water to the ZnO core.

Silica NP and shells formed by the Stöber process have a certain intrinsic porosity, which allows water and oxygen to pass at a limited rate (the diffusion coefficient of water being ten times lower than for unhindered diffusion [36]). Only silica layers with a thickness of about 100 nm or more were expected to be completely impenetrable for water and oxygen and would reliably block any chemical or photochemical reaction with the core material [37]. Although the resulting particles would be too big for applications as, e.g., UV blockers, it would be interesting to check the threshold shell thickness at which the degradation of the core by phosphate is no longer detectable; this is, however, beyond the scope of the current investigation.

XRD measurements of the partially transformed ZnO-NP after 3 h revealed, in addition to ZnO, the presence of two more crystalline phases, namely triclinic Zn_3_(PO_4_)_2_·4H_2_O (parahopeite), and Zn_3_(PO_4_)_2_·2H_2_O. No hopeite (orthorhombic Zn_3_(PO_4_)_2_·4H_2_O) was detected. In addition to amorphous zinc phosphate, hopeite and parahopeite were identified as reaction products from non-coated ZnO-NP and phosphate in sodium nitrate solution [24] (but no dihydrate), and hopeite and dihydrate during the precipitation of zinc phosphate in cell culture media from ZnCl_2_ and phosphate solutions [33] (but no parahopeite).

Since our control experiments with water instead of phosphate buffer did not show any obvious change in size or morphology of the ZnO-NP (both coated and uncoated), it is not the equilibrium ZnO + H_2_O ↔ Zn^2+^_aq_ + 2 OH^−^ alone that is responsible for the dissolution of the NP. Only after diffusion of the Zn^2+^_aq_ ions through the pores to the exterior with high phosphate concentration, precipitation of zinc phosphate starts. This process is slower for coated particles than for uncoated particles for which a direct contact of phosphate ions with the ZnO surface is possible. This may account for the almost exclusive formation of crystalline zinc phosphate. The difference to the observed phases by others remains, however, unexplained.

## Conclusion

Most nanoparticles are dynamical systems in aqueous solutions since equilibria between the solid state and the solution exist. This is in particular important for oxidic particles such as SiO_2_. Among the technically applied nanoparticles, zinc oxide is probably the most sensitive material in this respect. While the solubility in pure water is limited, one must carefully adapt the environment to its comparatively high reactivity. We could show that phosphate ions are able to degrade ZnO-NP regardless if coated by silica or not.

The results are of importance for any investigation of the interaction of ZnO-NP with biological systems and for toxicology studies when buffer solutions or cell culture media are applied [38–39]. It is essential to know the content of phosphate of these media before bringing them in contact with the ZnO-NP. For the rapid degradation of most of the particles a phosphate concentration of 100 mg/L (as in human blood) was sufficient. If no precautions are taken it is possible that the behaviour of zinc phosphate with respect to biological systems is determined instead that of ZnO nanoparticles. Some skepticism is also adequate for the interaction of particles having a zinc oxide shell with cells [40].

As a potential remedy for this situation, we suggest the use of β-glycerophosphate instead of phosphate in media and buffers. We found that ZnO-NP do not show any sign of attack by disodium β-glycerophosphate in a concentration of 2000 mg/L after one day. The reason is probably the much higher solubility of zinc glycerophosphate in water, compared with zinc phosphate, which does not lead to precipitation and equilibrium shifts. In the light of these results it seems essential to ensure a tight coating by a chemically resistant material before applying ZnO-NP for, e.g., medical or cosmetic purposes. Silica coating can suppress photocatalytic activity but cannot prevent destruction of the ZnO core. It remains to be determined if, e.g., a polyorganosiloxane coating performs better in this respect.

The presence of phosphate in the solution limits the concentration of Zn^2+^_aq_ since zinc phosphate precipitates rapidly. One could therefore consider its formation as a kind of detoxification. Small zinc phosphate particles might be transported to the liver for controlled recycling of the zinc ions [33].

## Experimental

*N*-(2,5-Bis(dimethylethyl)phenyl)-*N*'-(3-(triethoxysilyl)propyl)-perylene-3,4,9,10-tetracarboxylic acid diimide (MDPI) was prepared as described [26]. The successful fluorescence labelling of ZnO-NP was checked by irradiating the dispersions at 254 nm. Dry solvents (stored over molecular sieves 4 Å) were used in all cases. Solvents and reagents were purchased from Merck unless otherwise noted. TEM pictures were taken with a JEM 2100 F instrument (Jeol, Tokyo, Japan) on a carbon-coated copper grid (Plano, Formvar/coal-film on a 200 mesh net). The size of agglomerates was determinated by DLS measurements, together with the ζ-potential, with a Nano ZS instrument (Malvern Instruments), using dispersions of ca. 100 μg per mL. Powder XRD was performed with a Seifert 3003 TT instrument.

### Preparation of ZnO-NP under acidic conditions [28] (sample 1)

To a solution of 660 mg (3.0 mmol) zinc acetate dihydrate in 15 mL of 1-pentanol and 7.5 mL of *o*-xylene, 56 mg of toluenesulfonic acid monohydrate (Merck) were added and the mixture was heated to 140 °C under nitrogen for 4 h. After cooling to room temperature the mixture was centrifuged at 2500*g* for 40 min and the solid redispersed in 10 mL of ethanol and centrifuged again. This washing procedure was repeated two more times. From the mother liquors more product can be obtained by centrifugation at 13500*g* and application of analogous washing procedures. The total yield was 120 mg of ZnO nanoparticles (50%). For the labelled nanoparticles, 3 μmol (2.3 mg) of MDPI were added before the addition of the toluenesulfonic acid. The same workup led to a total of 90 mg (37%). All particles were stored as dispersions in ethanol. ζ-potential in water: −48.3 mV; labelled: −56.3 mV. DLS: 400 nm, unlabelled; 401 nm, labelled.

### Preparation of ZnO-NP under basic conditions [29] (sample 2)

To a solution of KOH (390 mg) in 50 mL of ethanol prepared at 60 °C under nitrogen were added 660 mg (3.0 mmol) zinc acetate dihydrate and 8 mL of methanol. After stirring at 60 °C for 30 min and cooling to room temperature the mixture was centrifuged at 2500*g* for 40 min and the solid was redispersed in 15 mL of ethanol and centrifuged again. The washing procedure was repeated two more times. The yield was 236 mg (95%). For the labelled nanoparticles, 3 μmol (2.3 mg) of MDPI dissolved in 8 mL of methanol were added instead of the pure methanol. The yield was 204 mg (83%). All particles were stored as dispersions in ethanol. ζ-potential in water: +47.3 mV; labelled: +60.7 mV. DLS: 144 nm, unlabelled; 309 nm, labelled.

### Labelling of commercial ZnO nanopowder (sample 3)

ZnO nanopowder was obtained from Sigma-Aldrich (≤1 μm, purity 99.9%). The powder (25 mg), 0.2 μg of MDPI and 2.6 mL of ethanol were placed in a glass vial (volume 4 mL) with a magnetic stirring bar and closed tightly by a screw cap with a teflon gasket. It was heated in an oil bath to 140 °C for 20 h under stirring. After cooling to room temperature the solid was separated by centrifugation (9500*g*, 20 min), redispersed in 1 mL of ethanol and centrifuged again. The washing was repeated two more times. The yield was 22 mg (88%). The particles were stored as dispersions in ethanol. ζ-potential in water: −32.9 mV. DLS: 436 nm.

### Labelling of commercial SiO_2_-coated ZnO-NP (sample 4)

A sample of Maxlight ZS-64 (Showa Denko, 77–82% ZnO, 18–23% SiO_2_) was labelled in analogy to the preceding procedure. The yield was 21 mg (84%). ζ-potential in water: −32.1 mV. DLS: 454 nm. Shell thickness (TEM) 3–6 nm.

### SiO_2_-coating of ZnO-NP [35] (sample 5)

To a stirred dispersion of 5 mg of labelled ZnO-NP (sample **1**) in 0.2 mL of ethanol, 90 mL of water, 5 μL of aqueous ammonia (25%) and 5 μL of tetraethoxsilane (TEOS, Sigma-Aldrich) was added. After stirring for 1 h, the dispersion was centrifuged (12000*g*, 30 min), redispersed in 0.5 mL of ethanol, centrifuged, then redispersed in 0.5 mL of water and centrifuged again. The resulting coated particles (4.8 mg) were stored in ethanol (1.0 mL). ζ-potential in water: −31.3 mV. DLS: 620 nm. Shell thickness (TEM): 4–5 nm.

### Reaction of ZnO-NP with phosphate

Buffer solutions of the Sørensen type were used at pH 7.0 with 100 mg/L phosphate (buffer A) and 2000 mg/L (buffer B). In addition, a solution of 2000 mg/L of disodium β-glycerophosphate pentahydrate (99.0%, Calbiochem) was used for comparison.

Zinc oxide nanoparticles (samples **1**, **2** and **3**, 1.25 mg each) were stirred with the buffer solutions (30 mL for buffer A, 1.5 mL for buffer B) or the β-glycerophosphate solution (4.9 mL) for the appropriate time (1 h or 24 h). The dispersions were then centrifuged at 13500*g* for 10 min. The solid was redispersed in 1 mL of water and centrifuged again. This washing was repeated two more times. The final product was redispersed in 1.5 mL of ethanol, and 0.1 mL were used for TEM measurements. From the remaining solution the ethanol was evaporated and the residue was dissolved in 10 mL of 0.01 N HCl and 10 mL of an aqueous solution of EDTA disodium salt (10 mg) for ICP-OES analysis according to DIN EN 11885 (Bayerisches Institut für Angewandte Umweltforschung und -technik (bifa), Augsburg). The results are presented in Table 1. In addition, the ζ-potential and the hydrodynamic diameter were determined in buffer B immediately after dispersion and after one hour of reaction, using dispersions of ca. 100 μg per mL. The results are shown in Table 2. XRD measurements were done on samples of ca. 20 mg of nanoparticles treated with buffer B (6 mL) for 3 h, isolation of the solids by centrifugation, and air drying.

### Degradation of SiO_2_-coated ZnO-NP by phosphate

Silica-coated ZnO nanoparticles (samples **4** and **5**, 2.4 mg each) were stirred with buffer solutions A (60 mL) or B (8 mL) for 1 and 24 h, respectively. After centrifugation (12000*g*, 15 min) the samples were redispersed in water (0.6 mL) and centrifuged again. This washing procedure was repeated two more times. The products were stored in ethanol (1 mL) and analyzed by TEM. See Table 2 for DLS and ζ-potentials. XRD measurements were carried out with samples of ca. 20 mg of nanoparticles treated with buffer B (6 mL) for 3 h, isolation of the solids by centrifugation (13000*g*) and drying in air.
